# Physician Associates in the Frame: Developing a National Competence Framework for Physician Associates Working in Mental Health

**DOI:** 10.1192/bjo.2022.137

**Published:** 2022-06-20

**Authors:** Pranav Mahajan, Helen Crimlisk, Ellie Wildbore, Paris Tatt-Smith, Tony Roth

**Affiliations:** 1 Health Education Yorkshire & Humber, Sheffield, United Kingdom; 2 Royal College of Psychiatrists, London, United Kingdom; 3 Sheffield Health and Social Care NHS Foundation Trust, Sheffield, United Kingdom; 4 Royal College of Psychiatrists, London, United Kingdom; 5 National Collaborating Centre for Mental Health, London, United Kingdom

## Abstract

**Aims:**

Physician associates (PAs) are becoming more commonplace in psychiatric services in the UK to help address long term workforce difficulties. The 2019 NHS Long Term Plan detailed a commitment to transforming mental health care in England recognising that services were not meeting current or future increase in demand. Health Education England's (HEE) report, Stepping Forward to 2020/21: The Mental Health Workforce Plan for England, described a longer-term strategy to expand the mental health workforce, including recruiting 5,000 people into ‘new roles’ including physician associates. The NHS Mental Health Implementation Plan 2019/20–2023/24 stated an aim of recruiting 140 PAs to the workforce over five years in addition to the requirements specified in the HEE report. Competence frameworks make the link between evidence and practice and can be a valuable basis for training, an agenda for supervision and a guide for self-monitoring and personal development for people working in the role.

**Methods:**

The competence framework was developed by the National Collaborating Centre for Mental Health (NCCMH). The work was overseen by an expert reference group, comprising experts in training PAs in mental health, PAs, researchers and experts by experience, all selected for their expertise in research, training and service delivery. The completed framework was then sent to relevant stakeholders including the Faculty of Physician Associates and patient groups for comment and adapted accordingly.

**Results:**

The completed framework has been arranged into seven domains: Knowledge of Mental Health, Professional/Legal Issues, Engagement and Communication, Diagnostic Assessment and Treatment Planning, Interventions, Team Working and Metacompetences. This reflects the expected roles and responsibilities of PAs working in mental health.

**Conclusion:**

The Competence Framework for PAs will help those involved in mental health care services who wish to deepen their understanding of the PA role, and will be useful to team members working with PAs, to their managers and to commissioners. It will support the work of PA supervisors and peer coordinators, and those delivering education and training to them. It also brings a level of standardisation of the role. More work will be needed to adapt the Competence Framework for PAs for specialist contexts, such as in dementia care or children and young people's services.

